# The Rise of *Patient Safety-II:* Should We Give Up Hope on *Safety-I* and Extracting Value From Patient Safety Incidents? Comment on "False Dawns and New Horizons in Patient Safety Research and Practice"

**DOI:** 10.15171/ijhpm.2018.23

**Published:** 2018-03-07

**Authors:** Andrew Carson-Stevens, Liam Donaldson, Aziz Sheikh

**Affiliations:** ^1^Division of Population Medicine, School of Medicine, Cardiff University, Cardiff, UK.; ^2^London School of Hygiene and Tropical Medicine, London, UK.; ^3^Usher Institute of Population Health Sciences and Informatics, The University of Edinburgh, Edinburgh, UK.

**Keywords:** Adverse Events, Preventable Harm

## Abstract

Who could disagree with the seemingly common-sense reasoning that: "We must learn from the things that go wrong."? Despite major investments to improve patient safety, relatively few evaluations demonstrate convincing reductions in risk, harm, serious error or death. This disappointing trajectory of improvement from learning from errors or Safety-I as it is sometimes known has led some researchers to argue that there is more to be gained by learning from the majority of healthcare episodes: the things that go right. Based on this premise, socalled Safety-II has emerged as a new paradigm. In this commentary, we consider the ongoing value of Safety-I based approaches and explore whether now is the time to abandon learning from "the bad" and re-energise data collection and analysis by focusing on "the good."

Internationally, it is now widely acknowledged that healthcare-associated harm poses a major threat to public health and wellbeing.^1^ As long ago as 2002, the World Health Assembly called for urgent action to address this problem.^2^ Almost immediately, a number of health systems responded to this call to arms, which involved having at the core of their mission a drive to learn from medical errors and adverse events through patient safety incident reporting and learning systems. The assumption was that such a process would identify systemic weaknesses that contributed to the error(s) and capture the learning from these, which could then be used to prevent future recurrences. Who could disagree with the seemingly common-sense reasoning that: “We must learn from the things that go wrong.”? Each incident report would be “a window on the healthcare system.”^3^ Moreover, the improvements in other safety critical industries owe much to the analysis of data from reports of incidents – both near misses and accident investigations.^4-6^

Yet, major investments to establish patient safety have yielded relatively few evaluations that have demonstrated convincing reductions in risk, harm, serious error or death. This disappointing trajectory of improvement from what has been characterised as *Safety-I* has led some researchers to call for different thinking.^7-9^ Instead, it has been argued that there is more to be gained by learning from the majority of healthcare episodes: the things that “go right.” From this position, so-called *Safety-II* has emerged as a new paradigm. Descriptions of the role of *Safety-II* in health care improvement have been judged as “confused or overlapping, or are ambivalently expressed.”^10^ In this *Journal*, Mannion and Braithwaite propose a more nuanced, compromised position where *Safety-I* and *Safety-II* approaches coexist in the interests of maximising insights to support health systems improvement.^11^

## Achieving Analytical Primacy – Four Challenges for Safety-II

Proponents of *Safety-II* have a strong case for a paradigm shift in preventing harm. At the outset, though, to achieve analytical primacy, *Safety-II* has to address four key concerns. First, that the generation of harm in healthcare, through system failure, is similar to other sectors. These sectors have used learning from this perspective to good effect.^4,12^ Second, growing expertise within a small number of research groups worldwide is yielding powerful insights into the causes of preventable harm, which will we believe in time lead to the development of interventions that can then formally be evaluated. We take heart from examples like the PINCER trial, a pharmacist-led, information technology-based intervention used to minimise a range of medication errors. PINCER demonstrated that it is possible to move from insights from descriptive and qualitative work to developing medication safety interventions which are then shown to be clinically effective and cost-effective.^13,14^ PINCER is now being rolled-out as a routine implementation study across a much larger population base. This work needs more support. Third, the harrowing, unsafe care experiences of patients and their families depicted in incident report databases represent a unique perspective for learning and should not be overlooked. The volume of data in many is now so great that much have never been analysed or used to support improvement.^15,16^ Organisations are hindered by lack of investment for building capacity and capability of staff to analyse such data.^15^ The public would surely be horrified to learn of this inability to honour those that have suffered. Healthcare leaders should not be comfortable about this. To not even bother to look at a story of a patient’s experience of harm inflicted by the health system is a long way from the core value of respect. Finally, given the very significant data analysis challenges posed by the very high volume of patient safety incidents now being reported, we need to be very wary of trying systematically to analyse and learn from and feedback to reporters on the very high number of episodes of care that go right. In relation to this last point, our argument is based on pragmatic considerations.

## Lessons Learnt From Safety-I

Following the report of a public inquiry into a wholesale failure in standards of care in an English hospital, the founder of the Institute for Healthcare Improvement, Donald M Berwick was asked by the United Kingdom government to conduct a review of patient safety. In his report, *A promise to learn - a commitment to act: improving the safety of patients in England*^17^ he highlighted that “organisational learning is key to improving patients’ safety.” This echoed previous recommendations made in *An Organisation with a Memory*^18^ over a decade earlier, and suggests the National Health Service (NHS) had been slow to realise how to generate and act on learning from healthcare-associated harm.

Berwick stated:

“*Organisations should demonstrate that they have in place fully functional reporting systems for serious incidents, that staff know how to use them, that the systems are used, and that appropriate action is taken in response to incidents, including provision of appropriate support to the affected patients and their carers.”^17^*

Epidemiological studies of advanced healthcare systems find the same systemic failures and sources of healthcare-associated harm – for example, surgical complications, falls in healthcare facilities, medication errors, pressure ulcers, and others – recur year after year in many countries.^19^ Incident reporting systems have contributed a reveal on how systems could be designed to minimise future risk to patients. For example, a third of patient safety-related hospital deaths in England involved poor management of the deteriorating acutely ill patient,^20^ sometimes called “failure to rescue.” Similarly, learning from primary care reports has highlighted options for error-proofing the design of systems in general practice,^15^ between primary and secondary care,^21^ and highlighting risks to specific patient groups including older adults and children.^22-25^

The difficulty with *Safety-I* is not that it has held back an understanding of unsafe systems. It is that few reliable ways have been found to strengthen how the learning is used to improve the healthcare system in a way that sustains a risk reduction. There have also been limited opportunities, and in some respects ability, to undertake detailed investigations of incidents of the kind that are needed to help develop interventions. Whilst research groups have provided some insights to help decide which incidents to focus attention on, many have been reluctant to take the difficult step of moving to intervention development and testing. We recognise doing this kind of research is challenging, largely arising from the relative infrequency of actual harm such that surrogate measures are often needed, which do not appeal to funders or high impact journals.

## Reporting and Learning Systems for Safety-I and -II

Reporting systems can be used to identify trends and patterns of avoidable incidents and their causes (including near misses), opportunities to develop evidence-based models for safe practices and support for education and learning. Similarly, they have potential to capture learning from episodes of peer-reported excellence or positive deviance.^26^ However, quality improvement, informed by rigorous analysis from local patient safety incident management systems, are the exception. Too much data are collected and too little are done with it.^27^ This apparent lack of demonstrable progress probably deters reporting since few staff see the rewards of their conscientiousness in trying to protect patients. Not closing the feedback loop to incident reporters has in some cases led to frustration and disillusionment. An open reporting culture that staff can trust, and have confidence their concerns will be acted upon, is a prerequisite to building safer care.^4,17^

Our efforts to tackle patient safety in primary care have been enriched by lessons from our analysis of over 60 000 patient safety incident reports. We have come to learn that the benefits of incident report analysis are supported by a structured analysis of free-text information about what happened, perceived contributory factors and actions to prevent future occurrences. To achieve this, we developed a taxonomy aligned with the World Health Organization’s (WHO’s) International Classification for Patient Safety to support future global knowledge sharing, and our method for generating learning is now used in six countries.^15^ Crucial to our success has been the application of the *Recursive Model of Incident Analysis, developed by the Australian Patient Safety Foundation,*^28^ for organising our analysis and identifying salient relationships in coded data.^15,21,23-25^ Our diagnostic approach has generated multiple recommendations for quality improvement in primary care (as quality improvement plans in a format called a driver diagram),^21,22^ and is informing the design of a national primary care patient safety improvement agenda in Wales.

From gaining clarity on definitions of patient safety incidents in primary care, we produced Royal College of General Practice “how to” guidance and e-modules to support primary care teams to identify, report and learn from patient safety incidents, as well as suggestions on how to include patients and families in this process.^29,30^ In this guidance, we also advocate that the analysis of incident reports be an essential step in ‘diagnostics’ when planning a quality improvement project. A similar trajectory of work is needed to support staff to report excellent care. This should be the case right across the healthcare continuum and organisations should be asking how analysis of incident reports can regularly inform their improvement agendas. One organisation in Wales, United Kingdom, used our methods to analyse patient safety incident reports about anticoagulation error. The subsequent improvement project demonstrated fewer delays in discharge, hospital acquired pneumonias and other complications, and created demand for legislative amendments to The National Health Service (Wales) Act to put systems into practice to improve anticoagulation safety across the country.^31^

- Based on our experience of research using patient safety incidents, particularly in primary care, we believe an *incident reporting system should:*
- Be designed to understand why unsafe (or near miss), suboptimal, or excellent care occurred;
- Use the WHO Minimal Information Model,^32^ particularly to ensure reporters describe what happened, perceived contributory factors and the patient outcome;
- Permit identification of priority concepts for improvement that minimise or prevent safety risk (*Safety-I*) and bolster the positive processes (*Safety-II*) that matter to the workforce and to patients;
- Raise hypotheses for research, particularly informing the design of interventions for testing (moving from feasibility to pilot trials and eventually full trials) and implementation (using quality improvement methods), as well as be used to corroborate insights from existing research studies; and,
- Use an existing internationally accepted conceptual model for understanding patient safety, the WHO International Classification for Patient Safety,^33^ to allow global sharing of data for exchange of solutions for common areas of challenge and maximise opportunities to learn from rare events.

## Conclusion

If used to their full capacity, and crucially if they are subject to further development in analytical methods and tools, we believe that patient safety incident reporting and learning systems have a crucial part to play in making healthcare safer. Powerful exemplars of improvement driven by local and national incident reporting systems demonstrate their potential. Healthcare leaders, and their organisations, must be responsible for developing robust mechanisms to ensure patient safety incident reporting systems capture essential information that can inform improvement efforts, be systematically interrogated and used to redesign care processes. Frustration with their lack of impact has helped fuel the concept of *Safety-II*. We agree with the latest articulation of *Safety-II* in that there is also a need to learn from what goes well, ie, safe episodes of care, is important. Leaders should proactively and simultaneously seek signals for improvement from unsafe, suboptimal and excellent care.

## Ethical issues

Not applicable.

## Competing interests

Authors declare that they have no competing interests.

## Authors’ contributions

All authors contributed equally to the writing of this article.

## Authors’ contributions

^1^Division of Population Medicine, School of Medicine, Cardiff University, Cardiff, UK. ^2^London School of Hygiene and Tropical Medicine, London, UK. ^3^Usher Institute of Population Health Sciences and Informatics, The University of Edinburgh, Edinburgh, UK.
